# Supplementary figures and images for: Dynamic neurogenomic responses to social interactions and dominance outcomes in female paper wasps
Source: PLoS Genet. 2021 Sep 3;17(9):e1009474. doi: 10.1371/journal.pgen.1009474 (PMC8415593; doi:10.1371/journal.pgen.1009474)

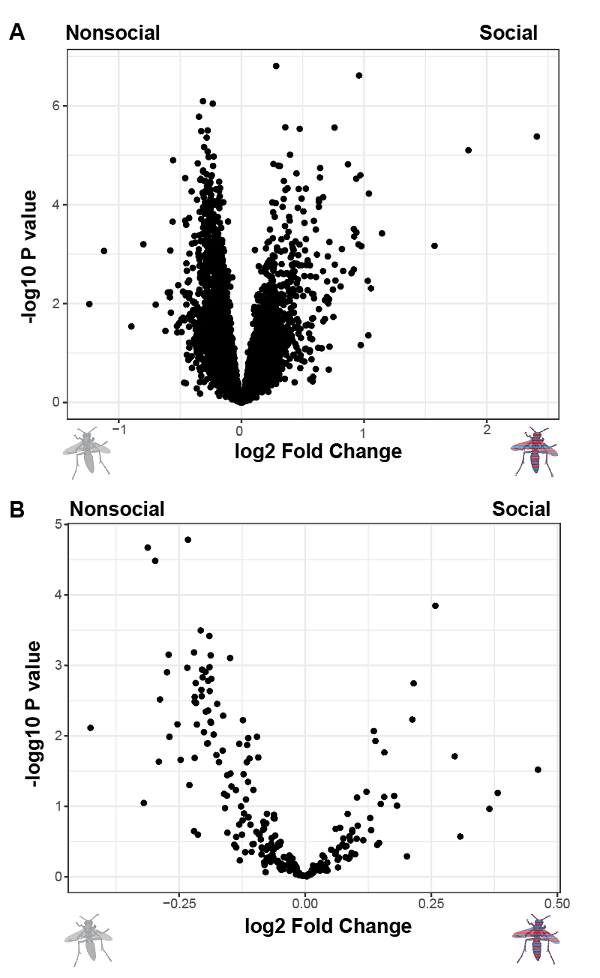

Supplement: S1 Fig — The volcano plots show the genes that are differentially expressed between individuals that experiences social interaction (social) versus those that did not (nonsocial). Higher log2 fold change values indicate upregulation in the social group compared to the nonsocial group. Panel A shows the data for all genes examined. Panel B shows the plot for genes annotated with memory-related functions. The red and blue striped wasp symbol indicates that the data includes all socially interacting wasps. (PNG) [file pgen.1009474.s001.png]

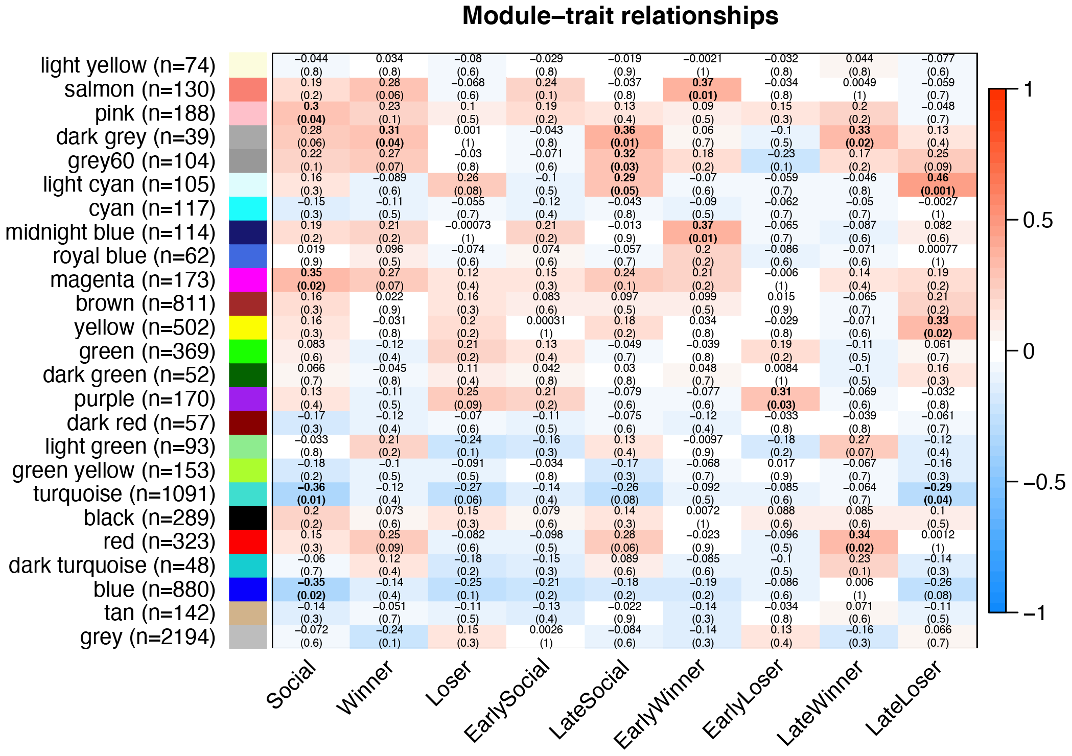

Supplement: S2 Fig — The heatmap shows the relationships between the modules of co-expressed genes identified by WGCNA with different social outcomes. White and lighter colors indicate low correlations. Bolder reds indicate stronger positive correlations. Bolder blues indicate stronger negative correlations. In each cell the top line of text reports the correlation between a given module and the social trait or time since social interaction. The bottom line of text shows the P value in parentheses. Significant correlations (P ≤ 0.05) are highlighted with bold text. (PNG) [file pgen.1009474.s002.png]
