# Supplementary material for: Dynamic neurogenomic responses to social interactions and dominance outcomes in female paper wasps
Source: PLoS Genet. 2021 Sep 3;17(9):e1009474. doi: 10.1371/journal.pgen.1009474 (PMC8415593; doi:10.1371/journal.pgen.1009474)
Supplement: Attachment — Submitted filename: ReviewerResponses.Uyetal.PlosGenetics.final.1.docx [file pgen.1009474.s018.docx]

Reviewer #1: How the outcomes of social interactions affect future behavior through the brain remains an area of great interest to sociobiology. Here, the authors use the primitively eusocial paper wasp *Polistes fuscatus* to examine the effect of a social challenge and the valence of its outcome on the global transcriptional state of the brain during the minutes and hours after these interactions.

The authors take advantage of the interesting biology of these paper wasps, who can recognize individual conspecifics, to differentiate the immediate and later effects of social challenge on the optic lobes and central brain. In general, this is a well-designed and well-powered study. The bioinformatics and statistical techniques used to analyze the RNA-seq data were appropriately chosen and applied. The authors have made their data available and say they have made their code available according to the standards of the genomics field. Overall, this is an excellent addition to the budding field of comparative genomics of social behavior. Figure 5, in which differences manifest in expression between winners and losers, is a particularly important contribution.

R: Thank you for the encouraging comments – and we agree that Figure 5 (now Figure 6) is a striking and helpful way to understand some of the changes that are taking place after the interactions have happened. Seeing the data plotted out that way certainly influenced our understanding of the overall dataset.

My comments are relatively minor and mostly confined to issues of dataset presentation.

* I can’t access the authors’ code. I’m not certain if they’ve uploaded it to the journal website and it can’t be accessed because of limitations with the editorial manager or if they haven’t yet made the code public on a resource such as GitHub. Please make sure the code is accessible on publication.

R: Thanks for catching this file upload issue. The R code has been uploaded - successfully this time – as a supplemental file.

* In Lines 162-183, the authors state that the PCA does not strongly separate social from non-social samples. However, the results of the PCA are only reported for the first two principle components. Have the authors examined subsequent principle components? What proportion of variance is explained by PCs 1 and 2 versus subsequent PCs?

R: We have now expanded the discussion of the PC analysis to address these questions as well as similar questions raised by another reviewer (see **lines 212-228**). We have redone the PC analysis using slightly different approach compared to our initial submission, so some of the particular numbers are changed, but the same overall story remains true. We do not observe clear separation between wasps that had a social experience versus those that did not, though we see a clear visually apparent separation between the optic lobe and non-visual brain in the first PCs. The first two PCs explain 41.77% of the variation (PC1=27.75%, PC2=14.02%). We examined a series of linear models with tissue, social interaction, and time of sacrifice as categorical predictor variables, as well as all two-way interactions. In these models, we find the samples significantly differ between PC1 and PC2 based on tissue. PC1 is also significantly influenced by whether oor not wasps had engaged in a recent social experience. PC5 (3.5% of variation) is differentiated by time and social behavior as well. Other PCs that are significantly related to tissue, social behavior, or time explain less than 2% of variation. We stopped investigating the variables that explain variation in PCs at PC15.

We investigated the dataset using PCA further in two ways. First, we subset the output from the PCA of all samples to ask how samples within each tissue type were influenced by social interaction, time, and their two-way interaction. In these analyses, we find that that PC1 and PC5 are influenced by social interaction for the non-visual brain samples, but not the optic lobe samples. The largest PC influenced by social behavior in the optic lobe data is PC10 (1.34% of variation). Second, we conducted separate PCAs on the central brain and optic lobes samples respectively. In the central brain PCA, the largest PC significantly different between social and non-social wasps is PC3 (7.59% of variation). PC1 (23.86% of variation) has a marginally significant association with social behavior as well (P = 0.09). For the optic lobe PCA, the largest PC significantly different between social and non-social wasps is PC7 (2.87% of variation).

Collectively, these analyses reinforce the interpretation in the initial manuscript that the social interactions in our study did not cause markedly distinct brain states between those that did or did not engage in a social interaction. However, there are detectable effects of social experience among the largest PCs. Importantly, these effects have a stronger impact on the transcriptomic profiles of the brain compared to the optic lobe.

* Lines 223-234: a false discovery rate correction should be applied to the Gene Ontology enrichment results to account for multiple testing. As they have 61/742 gene sets at a raw p < 0.01, I believe the authors will still find plenty of evidence for overrepresented GO terms after FDR correction is applied. A threshold such as FDR < 0.10, which the authors used for differential expression, would be appropriate.

R: Thanks for raising this point. Initially, we had not performed an FDR correction because we viewed the GO term enrichment analyses as a way to explore the data and thus it seemed reasonable to us to simply report a values below modestly low Pvalue threshold (0.01), an approach we had seen in the literature. The reviewer’s question, however, prompted us to look into the issue further, which revealed an unexpected level of complication. The test we used ‘weight 01’ in TopGo determines the Pvalue for a given GO term based in part on the values of related GO terms (i.e. weighting the results). As a result, the statistical tests are not independent and so the application of FDR based approaches is less straightforward. The authors of the TopGO package discuss this issue in the packages vignette and in fact argue against the use of FDR approaches in examining the list of results. Nevertheless, we appreciate that readers may not be familiar with these issues (as was the case of us) so we have added text in the methods section briefly discussing why we did not provide and FDR correction (**lines 704-707).** We do, however, provide a supplemental table with the outcomes of the tests so that readers can see the level of support for different GO terms for themselves.

* The paragraph in lines 316-331 (WGCNA results) contains some verb tense inconsistencies with the paragraphs flanking it. Please revise to use a consistent verb tense.

R: Thanks for pointing this out. We have now gone through the paper and made an extra effort to look for tense inconsistencies. We describe what we did or what others found in past tense throughout the paper. We describe relationships between data or other factual information in present tense.

* Line 549: I’m assuming that the threshold of average expression below 100 means that they filtered out genes with an average of 100 counts per sample or less. Please label units to clarify.

R: Yes, we removed genes with low normalized average read counts from the DESeq2 output for this analysis. The reason for this is that we compared log2 fold change between the winners and losers and log2 fold change levels are sensitive to expression level (this is commonly seen in MA-plots in a range of RNAseq studies where low expression genes show a large amount of noise in log2 fold change levels). The goal of this filtering was to reduce the noise associated with low-expression genes. The filtering was done on output of DESeq2 rather than input. This is now discussed more clearly in the methods section (**lines 670-676**).

* Lines 563-568: though it should be possible to find this information in the code associated with the paper, please give more details about the parameters used for WGCNA. Specifics that should be included are: which correlation was used (mid-weight bicorrelation or Pearson)? How was the soft threshold established? Were coexpression modules constructed using signed or unsigned correlations? How many genes were in the grey (unclustered) module?

R: Thanks for pointing out the lack of clarity in these methods, which we have now expanded (**lines 689-696**). We report the Pearson correlation for the correlation matrix. The soft threshold power (10) was established by plotting the power across threshold values from 1-20. The number was chosen based on where the value plateaued. We used a signed correlation matrix. There were 2194 genes in the unclustered (grey) module. Based on the comments of another reviewer we have modified the output of the supplemental figure showing all the correlation values to show how many genes were in each module.

* Figure 3: the Venn diagram panel does not use areas scaled to the size of gene sets in each bin. The authors should re-make this panel to present the gene sets as scaled. A suggested R package is VennDiagram, which has facilities for scaled Venn diagrams (see Figure 1B in PubMed ID: 21269502).

R: Scaled diagrams with three circles are often hard to interpret visually and we found this to be the case for our data as well. In particular, it made it challenging in our opinion to see the smaller amounts of overlap. The numbers provide the overlap sets and inform the reader of the relative magnitude of overlap. For what its worth, the vignette for VennDiagram notes “General scaling for three-set Venn diagrams are disabled due to potentially misleading visual representation of the data.” We have added a line in the figure legend (now figure 4) that notes that the circles are not scaled (**line 995**).

* Figure 4C: it would be helpful if the colors of the bounding boxes around the dendrogram meta-modules and the colors of the bounding boxes around the heat map meta-modules matched.

R: Thanks for this suggestion. Unfortunately, the red and blue colors used for denote winning/losing and that are also used for the heatmap mean that the bounding boxes being red or blue make them rather hard to see. However, the names of the wasp categories (late loser, late winner) are color-coded and the regions line up vertically in the figure. Since the black lines are more visually apparent in the heatmap, we have kept the figure as it was originally displayed.

* A supplementary table containing the gene IDs of all genes highlighted in red, blue, or purple in Figure 5A would be helpful to others interested in this work.

R: We have added a supplemental table that provides details of all of the highlighted genes.

* Figure 5B please label the unit of measurement (CPM, TPM, RPKM?), preferably in the Y axes of the figure panels.

R: The Y-axis reports normalized counts as output by DESeq2. This is now reported more clearly in the figure and the figure lengend.

Reviewer #2: In the manuscript “Dynamic neurogenomic responses to social interactions and dominance outcomes in female paper wasps” Uy et al. describe the brain transcriptomics responses to competitive social interactions in female paper wasps, Polistes fuscatus, The authors find that subordinate wasps are less aggressive, forage more, and lay fewer eggs compared to dominant foundresses. They also find considerable differences in brain gene expression, including those related to aggression, worker behavior, and reproduction. Overall, this is an interesting study that will make a valuable contribution. I have a number of comments and suggestions that will hopefully allow the authors to improve the manuscript further.

General comments:
1) Both the Introduction and Results/Discussion are difficult to follow and can be structured more clearly. The authors start out with a big question (“How are social outcomes translated into physiological changes?”), and then meander through different topics in a way that makes the reader’s head spin. It is not until the final paragraph of the Introduction that the specific questions asked in this study become clear. Similarly, the presentation of the Results can be streamlined. Finally, I think the paper would be more compelling if the Discussion would be separate from the Results.

R: Based on these comments we have reworked the organization of the manuscript considerably. First, we have shortened to introduction with the aim of a more concise introduction being easier for readers to follow. Second, we have placed the discussion in a separate section following the results rather than as a combined results/discussion section. These changes have helped streamline and better organize the manuscript.

2) I understand the rationale for examining the optic lobes and “central brain,” though given the repeated emphasis on cognitive and similar higher order processing, I wonder whether the authors considered analyzing gene expression in the mushroom bodies.

R: We had considered looking at specific neuropils, such as the mushroom bodies, but ultimately decided against it for a few reasons. First, it is technically more challenging to extract sufficient RNA from smaller volumes of tissue so we were concerned about losing too many samples. Given that we had some samples drop out even with larger tissue blocks, this concern seems to have been reasonable (which isn’t to say it would be impossible). Second, while there are a growing number of studies of brain gene expression in social insects, precisely what tissue has been sequenced in neurogenomic studies among social insects has been variable (e.g. head, whole brain, non-visual brain only, just the mushroom bodies, etc). While the goal of our paper is not to directly compare to other studies, we were cognizant of the potential for future comparisons across datasets so modeled our dissection after similar work that has been done in Harpegnathos ants (Gospocic 2017 *Cell*, Opachaloemphan et al (2021) *Genes and Development*), though unlike in those studies we also sequenced the optic lobe, since visual behavior was of particular interest to us.

In future studies, we would like to examine more specific neuropils, though with the decreasing costs of single cell sequencing we could imagine that examining individual cell-level responses to social interactions may be the most useful approach moving forward.

3) Provide more information on the behavior data. For example, consider showing representative timelines of behavior (one aggressive act every 1.5 minutes seems low?) and graphs that summarize behavior for winners and losers (along with appropriate statistical analysis). This would also help the reader to get a better understanding of what a “more vigorous encounter” (line 565) is as opposed to most trials being “comparatively mild” (line 181; compared to what?), and why only social trials with at least 10 aggressive acts were analyzed (line 543).

R: We have added information about the behavioral trials (**lines 182-206**) as well as provided all of the scored behaviors as a supplemental file. We also clarified that the trials (as is similar to other trials in neutral arenas) are relatively mild in the scheme of aggression that wasps are known to engage in (when defending a nest, foundresses can engage in lengthy battles and falling fights that may end in death – interactions in neutral arenas are much milder).

4) The presentation of the PCA results can be improved. Consider showing scree plot (to show how variance components break down) and box plots with statistics for PC1 and PC2; providing information on whether higher order PCs separate any of the experimental groups. Because most of the variance is explained by brain region (as expected), I suggest conducting PCA for each brain region separately as well.

R: We have redone the PCA analysis in conjunction with these comments and the comments from Reviewer #1. In both the analysis of all the data (i.e., both tissues) or the analysis of each tissue type separately, we find that larger (and thus more explanatory) PC axes are associated with recent social interactions in the non-visual brain compared to the optic lobe.

5) The authors used DESeq2 for DEG analysis. Given the experimental design, limma might be more appropriate.

R: Thanks for raising this question as the diversity of methods available for analysis of RNAseq can be a bit overwhelming. Schurch et al (2016, RNA) compared multiple different RNAseq analysis pipelines. They suggest that limma is especially useful with large numbers of samples, but that DESeq2 is preferred when there are small numbers of samples per condition. While there are a large number of samples overall in our study, there are relatively few samples per condition (e.g. early time point winner central brains) so we have used DESeq2. That said, we appreciate that analysis methods can give different results, which is why we are reassured by the fact that we find similar patterns regarding winners and losers from analyses using DESeq2 and WGCNA, which have very different assumptions and approaches.

Specific comments:
Line 52: Provide a reference for physiological response to cooperative interactions.
- Line 67f: See also the recent study by Vu et al. (<https://doi.org/10.1371/journal.pgen.1008831>), which is cited later on.
- Lines 282-285: Consider moving these sentences before line 277.
- Line 317: WGCNA stands for weighted gene co-expression network analysis, not “correlation network analysis.”

R: We have made these changes where appropriate, though a sufficient amount of the introduction was re-worked so not all specific recommendations fit the new version.

- Line 319: Provide number of genes for each module (this info could be added to figure 4C)

R: This is a great suggestion. We’ve added the numbers to a supplemental figure (Fig S2) that has more space and was easier to see.

- Line 477: How long are the wasps in the lab before testing?

R: Details about collection, including date, have now been provided in a supplemental table

- Line 489: Consider rephrasing such that it is clear that each group has the same n = 15, as there are winners and losers for both sets of social wasps.

R: We have now added text to more clearly explain the number of wasps **(lines 150-164**). Additionally, we modified Figure 1 to explain the experimental design as well as added a table to show the number of each type of sample present in different analyses.

Line 512: What parameters were used with STAR?

We used the following command (which is now given in the methods):

STAR --genomeDir ~/STARgenome --runThreadN 30 --readFilesIn ‘filename_here’.fastq.gz --readFilesCommand zcat --outFileNamePrefix filename_here’_ --outFilterMultimapNmax 1 --outReadsUnmapped unmappe_a --outSAMtype BAM SortedByCoordinate

- Line 526f: Can you be more specific on the filter criteria used for removing genes? And why not use the same criteria for both PCA and DEG analysis?

R: We apologies for the confusion in how we had written the methods section. The exact same dataset was used to analyze PCA and DEG analyses. This is now stated clearly in the methods (**lines 670-676**). The only additional filtering that occurred happened *after* DEG analysis when we compared the patterns of gene expression between winners and losers. In that case, we filtered out genes with less than a mean of 100 normalized counts in the dataset, to remove noise of logFold2 changes in very lowly expressed genes from comparisons of logFold2 patterns.

- Line 544: It is common to use a log-fold cutoff, but I don’t think it is necessary in this case. This should be clearly stated, however.

R: Agreed. We have added text as well as citations to the methods to explain this point (**lines 663-668**).

Reviewer #3: This is an interesting study of the brain gene expression in paper wasps as a result of social aggression or social isolation. The time course and replication of this study are well in line with former studies, but the sequencing effort and data analysis presented here lack comparisons with other relevant literature.

Results and Discussion
Lines 162-164: No mention of experimental design or behavioral results are presented here making it difficult to understand sample sizes, focal groups or behavioral assays. Some inclusion of the sample sizes, experimental design, and behavioral results are needed here since this information is curiously omitted from the results.

LIne 171-172: here again the reader has no idea what social and non-social mean in this context. Some behavioral results above would improve the readability
Lines 176-178: again the reader has no ability to assess this and it would be more useful to explain the behavioral treatments and design for this work then to state they are similar to other studies. Expand on this to explain the number of wasps and numbers of assays.

R: We have corrected these issues with manuscript presentation and now have a clearer description of the experimental design and provide analyses of behavioral data (**lines 150-164, 180-206**). There is now a table providing the specific numbers of wasps per assay.

Line 214: needs more justification why optics lobes were dissected in this study versus rest of brain. Why optic lobes and not antennal lobes or mushroom bodies?

R: We have added the following text to the introduction (starting **line 139**):

Visual facial recognition is a notable feature of *P. fuscatus* from Ithaca, NY, so we were also interested in the relationship between visual processing and social interactions. Previous studies of morphology have suggested that the visual system may have evolved to facilitate individual recognition and visual brain regions are developmentally sensitive to social isolation. Additionally, analyses of selection in *P. fuscatus* identified visual processing genes as enriched as recent targets of positive selection in this species, likely in relation to social interactions and individual recognition

Methods

Line 476: How many wasps per assay amount to the 90 focal females? Were all nests from different nests or how was this handled? More specifics on the age, reproductive status and colony of origin are needed here.

R: We have provided an additional supplemental table with this information (Table S6-7).

Lines 493-498: This data should be included in the results. Currently there is no mention of aggressive behavior or statistical treatment of assays to follow how winner and losers and time course data were generated.

R: Additional details are provided regarding behavioral trials (see responses to other reviewers and response to one of your earlier comments) which include a new results section and figures.

Lines 507-510: sample sizes are explained here but not by treatment or time point. How did the loss of samples from 180 to 168 to 139 reflect in social vs non-social and 45 min vs 4 hour treatments. What are the sample sizes per treatment.

R: We have provided a table to show the final sample sizes for each analysis (Table 1).

Line 509: 1 milion reads per sample seems very low. What is the justification for this? Most studies use 30-60M reads per sample for RNAseq studies of this kind. Viewing Table S1 only 2-5M reads were used for this study which is an order of magnitude less than comparable work. What effect does this have on the results presented here?

R: We apologize for the lack of clarity in the initial methods section. There was an average of 5.15 million unambiguously assigned gene counts in our dataset, which is roughly equivalent to sequence depth of 30-60M reads using other RNAseq approaches*. We now explain more fully the consequences of 3’RNAseq approaches (**lines 599-602**), which have been developed to reduce library production and sequencing costs for RNAseq experiments while making differential expression analysis more straightforward and require fewer assumptions. 3’RNAseq captures the polyA tail of eukaryotic mRNA using magnetic beads and the RNA is then fragmented. The captured RNA thus repents the 3’ end of genes and that single fragment from each unique transcript is then prepared for sequencing, typically with single end reads. The short reads near the 3’ end of the gene are sufficient to unambiguously map the genes. Because each transcript is only by a single read, the read count is equivalent to the number of unique transcript molecules assessed in the experiment. Thus, much lower sequencing effort is needed. We have added a brief overview of 3’RNAseq’s lower sequencing needs in the methods section.

*More “traditional” mRNAseq approaches have sequenced entire transcripts which means that more sequencing depth is needed to fully coverage each transcript. This is also why many analysis approaches take into account transcript length, since longer transcripts need more sequencing depth to be covered to the same degree as short transcripts. Additionally, sequence depth does not equal gene counts because in addition to variable transcript length, not all of the sequences align to genes (though here we reported *aligned* and unambiguously counted reads). Thus, there isn’t a very straightforward 1-1 comparison that can be readily made, but it is clear that we have sufficient depth in our study.

On a related note, there is no explicit comparison of these results to any other studies in honey bee, mice or sticklebacks, etc. Is this because of the low sequencing coverage or what were hypergeometric test comparing gene lists from other studies not explored here? Are the genes identified here significantly similar to other studies?

R: As mentioned in the previous section the coverage level is more than sufficient for RNAseq analyses. This is an interesting suggestion for future work that is outside the scope of this study (which focused on dynamic gene expression shifts in a particular species). We did not compare our data to data from other studies as that would not inform the questions we outlined in our paper and represents a distinct level of analyses. We are aware that many studies have set out to compare the similarity/differences in transcriptomic responses across disparate taxa, however it is not true that similar types of RNAseq studies necessarily compare across taxa. For example, Vu et al (2020) *PLoS Genetics*, Saul et al (2017) *Genome Research*, and Opachaloemphan et al (2021) *Genes and Development* all consider transcriptional dynamic changes in response to social interactions in fish, mice and ants respectively and none compared their data directly to other species (though the group in the Saul et al 2017 paper had been previously involved in comparing transcriptomic responses among diverse taxa). Finally, given that the manuscript is already rather long and detailed, adding additional analyses that are not focused on the questions outlined in the manuscript is something we feel would detract from the presentation of the results related to the questions we have set out to answer.
