# Supplementary material for: Dynamic neurogenomic responses to social interactions and dominance outcomes in female paper wasps
Source: PLoS Genet. 2021 Sep 3;17(9):e1009474. doi: 10.1371/journal.pgen.1009474 (PMC8415593; doi:10.1371/journal.pgen.1009474)
Supplement: Attachment — Submitted filename: Social_nonsocial.RNAseq.revision2.respponseToReviewers.docx [file pgen.1009474.s019.docx]

Dear Prof. Stubbs:

We are pleased that the reviewers found the revisions to have improved the paper. It is really a credit to their thorough comments in the earlier draft. We have made changes to the presentation of the GO enrichment analysis as suggested by reviewer 1 and highlighted in the editorial decision. Additionally, we have gone through the manuscript and lightly edited for grammar and spelling mistakes we found in the earlier draft. We made some other small changes, which we feel improve the manuscript. First, we have now uploaded a short video clip of a wasp behavioral trial. Second, we have uploaded individual .csv files for supplemental tables to make the data and presentation more consistent with F.A.I.R. publishing practices, by avoiding the use of proprietary software formats such as Excel.

The decision noted that we needed to address the comments for reviewer 1 but did not mention the other reviewers, so we have not specifically addressed the comments of reviewer 3, which seem to be based on a misunderstanding of how 3’prime RNAseq (also called QuantSeq) approaches work and how their library preparation is distinct from other mRNAseq library preparation protocols (such as those studied in the paper they mentioned).

Sincerely,

Michael Sheehan

Reviewer #1: I am satisfied with all responses to my reviews except the one related to FDR correction on GO analysis. I’m sorry, but false discovery rate correction is no less important in this instance. The authors of TopGO are probably right that the Benjamini-Hochberg method is inappropriate for the highly dependent p-values derived from GO analyses. Their assertion in their whitepaper that tests are already FDR-corrected by dint of the weighted method is dubious. If the TopGO authors attempted to shepherd this statement through peer review, I would venture to guess that they would receive substantial pushback from the statistical genomics community that solved this problem 20 years ago.

An FDR-correcting procedure known as Benjamini-Yekutieli (2001) was developed as an extension of Benjamini-Hochberg to conservatively estimate FDRs in the presence of dependencies between p-values. Applying this correction procedure is as simple as using the function p.adjust(pvalues, method = "BY") in base R. I think it reasonable to request that the authors apply this B-Y FDR correction procedure to the GO results. However, if they choose not to apply this procedure and retain the text as is, I would request that they report p-values for all GO categories in Supplemental Table S4. Presently, just the top 75 findings are reported. This does not allow the reader the opportunity to see the p-value distribution of the whole test set, which is what is necessary to truly assess the output.

**Response:** We thank the reviewer for the clear explanation of the issue as they see it. The reviewer suggests two different approaches (1) report the BY fdr correction or (2) simply give the entire list of P values. In our response, we have tried to split the difference between these two possibilities. We now provide the full list of 3096 GO terms output by the weight01 test from TopGO as well as FDR corrected p values in the supplemental table. We note that the FDR correction we used is the Benjamini-Hochberg test rather than BY as BH is used more commonly in GO term enrichment tests and is less conservative.

P value correction using FDR or other methods is important when there are many hundreds or thousands of hypotheses being tested since we expect some portion of the findings to be false positive (as well as false negatives). We were specifically interested in testing the *a priori* hypothesis that social interactions would have a transcriptomic signature of memory (last paragraph of the introduction, aim #2). To assess the extent of the ‘memory’ signature in the data we used GO enrichment analyses, which tests all possible terms by default. Thus, our goal was not to see which terms ‘rose to the top’ as if the case in some application of GO enrichment, but rather to assess the evidence for enrichment related to memory. From the perspective of specifically testing the hypothesis regarding memory, FDR correction based on 3096 terms is highly conservative since the vast majority of GO terms are superfluous to the specific question of memory. Of course, we didn’t only test memory related GO terms, so we appreciate the reviewer’s point that FDR correction is appropriate when considering the statistical approach employed here. By providing all the of the GO enrichment output with Fisher’s exact test P values as well as the BH FDR correction, readers can assess the level of enrichment. After FDR correction, anesthesia resistant memory remains significantly enriched.

Ultimately, we see the GO enrichment analysis as a relatively minor part of the paper that we think is presented in a sufficiently cautious manner (the winner/loser changes are more interesting by far!). There is a clear *a priori* prediction from previous behavioral studies that memories are formed and so we should be able to detect a signature of memory at the transcriptomic level. We believe the analyses show that to be the case, though we are quick to point out that there are puzzling features of the ‘memory signatures’ that we detect. The discussion section about memory highlights this again and emphasizes that this data suggests it’s possible to learn more about memory using transcriptomics, but we need more data from a wide range of experiences to say anything more conclusive. Thus, we feel our presentation of the GO enrichment results is now more evenhanded and cautious after addressing the concerns of the reviewer.

In addition to modifying the supplemental Table so it has all the GO terms and the FDR corrected P values, we have also made some small changes to the text. (1) In the main results section on GO terms we re-emphasize that we were interested in specifically testing the *a priori* hypothesis that there would be a signature of memory-related genes in response to social interaction. (2) In the methods section, we provide a discussion of the ‘weight01’ procedure and as well details of FDR correction procedures in addition to our logic for reporting Fisher’s P values in the main results section of the text.
